# Factors Affecting Cardiovascular Physiology in Cardiothoracic Surgery: Implications for Lumped-Parameter Modeling

**DOI:** 10.3389/fsurg.2019.00062

**Published:** 2019-11-05

**Authors:** Joshua Kaufmann, Ethan Kung

**Affiliations:** ^1^Department of Mechanical Engineering, Clemson University, Clemson, SC, United States; ^2^Department of Bioengineering, Clemson University, Clemson, SC, United States

**Keywords:** multi-scale modeling, patient-specific modeling, hemodynamics, virtual surgery, surgical planning

## Abstract

Cardiothoracic surgeries are complex procedures during which the patient cardiovascular physiology is constantly changing due to various factors. Physiological changes begin with the induction of anesthesia, whose effects remain active into the postoperative period. Depending on the surgery, patients may require the use of cardiopulmonary bypass and cardioplegia, both of which affect postoperative physiology such as cardiac index and vascular resistance. Complications may arise due to adverse reactions to the surgery, causing hemodynamic instability. In response, fluid resuscitation and/or vasoactive agents with varying effects may be used in the intraoperative or postoperative periods to improve patient hemodynamics. These factors have important implications for lumped-parameter computational models which aim to assist surgical planning and medical device evaluation. Patient-specific models are typically tuned based on patient clinical data which may be asynchronously acquired through invasive techniques such as catheterization, during which the patient may be under the effects of drugs such as anesthesia. Due to the limited clinical data available and the inability to foresee short-term physiological regulation, models often retain preoperative parameters for postoperative predictions; however, without accounting for the physiologic changes that may occur during surgical procedures, the accuracy of these predictive models remains limited. Understanding and incorporating the effects of these factors in cardiovascular models will improve the model fidelity and predictive capabilities.

## Introduction

There are several factors that can cause significant changes in the cardiovascular physiology during cardiothoracic surgeries and patient management. For instance, prior to surgery, patients undergo the induction of anesthesia and remain under its influence in the intraoperative and early postoperative periods. Surgeries may also require cardiopulmonary bypass and cardioplegia to isolate and depress the heart. Complications may arise due to unforeseen circumstances, and patients may be treated with fluids and vasoactive agents to improve their hemodynamic state.

The implications of these factors on the fidelity of computational cardiovascular models are far-reaching. Current cardiovascular models, in particular lumped-parameter models, seek to simulate patient physiology to aid in clinical decision making; these models are constructed using primarily pre-operative clinical data and do not consider the events which may affect patient physiology during surgery (1, 2). Therefore, the aim of this work is to summarize the primary factors affecting the state of physiology during the acquisition of clinical data and in the postoperative environment, and discuss their implications on lumped-parameter cardiovascular modeling.

## Physiology Under Anesthesia

Several anesthetic combinations are used in surgery, each with significant and unique effects on the cardiovascular system. Currently, there is no universally accepted combination of anesthetic agents for cardiac surgeries. The choice of drugs is determined by the pathophysiologic state of the patient and the knowledge of the anesthesiologist. For initial hypnosis, inhalational agents or intravenous agents are typically used, while narcotics and muscle relaxants are used during periods of high stimulation such as intubation. The management of anesthesia is important in maintaining stable hemodynamics and an adequate blood supply to body tissues.

Volatile inhalational agents typically cause dose-dependent myocardial depression. Nitrous oxide is an inhalational anesthetic sometimes used in the pre-bypass period due to its rapid onset and elimination. It can be used to increase pulmonary pressures, and, to a lesser extent, depress contractility (although this is usually offset by the release of catecholamines) (3). However, it is not used during bypass due to its high blood solubility and propensity to expand air bubbles, increasing the risk of air emboli. Isoflurane is one of the most widely used volatile anesthetics due to its minimal cardiac effects as well as its ability to reduce cerebral metabolic oxygen requirements. It causes arterial blood pressure to decline, but preserves cardiac output (CO) due to an active carotid baroreceptor reflex which decreases systemic vascular resistance (SVR) and afterload (3, 4). Desflurane is in many respects similar to isoflurane, however, in increased concentrations, it may cause temporary sympathetic activity (5). Sevoflurane is the newest of the volatile anesthetics. It is able to reduce myocardial damage and preserve ventricular function. Similar to isoflurane, it also depresses cerebral metabolic requirements, protecting the brain from ischemia. Most volatile agents directly alter the body's natural temperature regulation mechanisms as well, causing a diminished threshold for cold responses such as shivering and vasoconstriction, which when paired with vasodilatory effects, commonly induces hypothermia (6). The cardiovascular effects of inhalational anesthetics are summarized in Table 1.

**Table 1:** Cardiovascular effects of inhalational anesthetics (3).

**Drug**	**Blood pressure**	**Heart rate**	**Systemic vascular resistance**	**Cardiac output**	**Cerebral flow**	**Renal flow**	**Hepatic flow**
Nitrous oxide	0	0	0	0	+	− −	–
Halothane	− −	–	0	–	++	− −	− −
Enflurane	− −	+	–	− −	+	− −	− −
Isoflurane	− −	+	− −	0	+	− −	–
Desflurane	− −	0 or +	− −	0 or –	+	–	–
Sevoflurane	–	0	–	–	+	–	–

Other types of anesthetic agents include intravenous agents such as Barbiturates, Ketamine, and Propofol. Barbiturates are occasionally used in cardiac surgery, acting to depress the metabolic demands of the brain and providing protection against ischemia. They are usually safe only in hemodynamically stable patients, making their use rather limited. Their use is associated with a decrease in blood pressure and an increase in heart rate (HR) due to vasodilation and decreased contractility (7). Ketamine is a hypnotic used to block neurotransmission to the brain. It has been shown to increase HR and mean arterial pressure (MAP) and is sometimes used in patients with depressed cardiovascular function such as those with congenital heart disease (8). Propofol is another hypnotic becoming a popular choice for anesthesiologists due to its fast metabolism, enabling the rapid emergence from sedation and early extubation (9). Propofol causes a dose-dependent decrease in HR and blood pressure due to decreases in contractility and peripheral resistance (10).

Opioids and Muscle relaxants are other types of anesthetic drugs, which may be administered to control pain and muscle movement, respectively. Muscle relaxants such as succinylcholine may result in abnormal heart beats, however both opioids and muscle relaxants cause very minimal changes to CO and blood pressure when compared to volatile or intravenous agents (11).

Although the current line of anesthetic drugs has been used for some time, there is an active search for agents with fewer adverse effects. One candidate is the inhalational use of Xenon. Other inert gases such as Argon have been found to have anesthetic qualities, but Xenon possesses the most potent anesthetic capabilities of the inert gasses. In an animal trial using Xenon on guinea pigs, Stowe et al. demonstrated that it had no mechanical, electrical, or metabolic effects on isolated guinea pig hearts (12). In a study comparing the effects of Xenon with propofol, Xenon sedation after cardiac surgery was found to be as safe as Propofol, while also offering an additional advantage of a rapid awaking time (13). Factors currently limiting the use of Xenon in cardiac surgery are its cost and the requirements for a unique delivery system.

## Factors Influencing Postoperative Physiology

### Cardiopulmonary Bypass and Cardioplegic Solution

Some operations such as valve replacement, congenital heart defect surgery, and coronary artery bypass graft (CABG) surgery require the use of a CPB machine to take over the function of the patient's heart and lungs, enabling the stopping of the heart. The heart is most often stopped by a cardioplegic (CP) solution decreasing the resting potential of the heart. This stops the heart, reduces its metabolism and oxygen demand, and allows the cardiac surgeon to work on a bloodless and motionless heart during the procedure.

The CPB machine operation and the CP solution directly impact postoperative states. Davidson et al. found that increased doses of CP were associated with greater degrees of increased left anterior descending coronary artery flow after congenital cardiac surgery (14). A meta-analysis of 5,879 patients comparing warm and cold cardioplegia found that warm cardioplegia was associated with a higher postoperative cardiac index (CI) but suggests other possible influences such as intermittent vs. continuous, antegrade vs. retrograde, and composition of CP (15). CPB perfusion temperature is another factor which has been found to influence postoperative patient conditions. Tönz et al. found that systemic vascular resistance at 3 hours and 9 hours after CPB was higher for patients who underwent surgery with hypothermic CPB as compared to normothermic CPB (16).

### Postoperative Complications

Typically, there is an increased oxygen demand as patients are weaned off CPB and anesthesia. There are similar increases in oxygen metabolism in both CABG and off-pump CABG patients, likely due to inflammatory responses to surgical trauma (17). The surgical stress response, whose magnitude and duration are proportional to surgical injury, increases activity of the sympathetic nervous system and causes a release of hormones which promote catabolism, increasing the overall metabolism (18).

A common complication following cardiac surgery is low cardiac output syndrome (LCOS). This is typically due to diminished heart pump function, leading to reduced oxygen delivery (DO_2_) and tissue hypoxia. It is characterized by a CI <2.0 L/min/m^2^ and a systolic blood pressure <90 mmhg (19). Physical signs of LCOS include tissue hypoperfusion resulting in cold and clammy skin, confusion, and elevated lactate level. Many other complications arise from LCOS such as acute renal failure, atrial fibrillation, and pulmonary complications. Common underlying mechanisms behind LCOS are left ventricular (LV) systolic dysfunction, LV diastolic function, and right ventricular (RV) dysfunction.

LV systolic dysfunction is commonly a result of myocyte necrosis due to impaired coronary circulation and ischemia/reperfusion injury. The ventricle's impaired response to preload (reduced ejection fraction) causes a decrease in CO and DO_2_ and increase in left atrial pressure (LAP). LV diastolic dysfunction is the inability of the left ventricle to accept an adequate volume of blood, despite a normal preload. This may be caused by severe tachycardia, decreased myocardial compliance, or impaired ventricular relaxation. Diastolic dysfunction is a common occurrence in cardiac patients in the postoperative period after CABG due to increased chamber stiffness (20). Right ventricular (RV) dysfunction, which may be developed due to intraoperative RV ischemia and infarction, is characterized by increased RV preload, increased RV afterload, right coronary artery perfusion impairment, and decreased RV contractility (19).

Another common postoperative condition occurring in 5–15% of patients following cardiac surgery involving CPB is vasoplegia, characterized by low systemic vascular resistance and high or normal CO (21). A lack of vascular tone and decreases in blood flow result in inadequate driving perfusion pressures to the tissue, reducing oxygen delivery and causing subsequent “shock.” Temperature and duration of CPB, volume of infused CP, preoperative treatment with angiotensin-converting-enzyme inhibitors, and reduced left ventricular function were found to be risk factors for vasoplegia (21).

Other complications which can lead to shock include hemorrhage and sepsis. Hemorrhagic shock is a type of hypovolemic shock produced by a rapid loss of intravascular blood volume. Rapid decreases in blood volume typically lead to decreases in CO and DO_2_ due to inadequate left ventricular preload. However, there is little change in VO_2_ due to a redistribution of blood flow to tissues with greater metabolic needs caused by the lowering of regional vascular resistance. As the volume of blood loss increases, blood pressure continues to drop, and heart rate increases in an compensatory effort to increase CO (22). However, when blood loss exceeds approximately 30% of blood volume, the sympathetic nervous system becomes inhibited, resulting in a normal or decreased heart rate and a drop in arterial blood pressure (23). In this sympatho-inhibitory phase, DO_2_ decreases to the point where VO_2_ can no longer be maintained.

On the other hand, sepsis is defined as the presence of systemic inflammatory response syndrome (SIRS) symptoms with suspected or proven infection. SIRS is a stress response which occurs as a result of injury such as that caused by surgical trauma or cardiopulmonary bypass, whose symptoms include abnormalities in body temperature, heart rate, respiratory rate, and white cell count (24). The initial SIRS response may lead to immunosuppression, making patients more susceptible to infection and the development of sepsis (25). Some of the diagnostic criteria for sepsis are: HR >90 beats/min. or >2 standard deviations (SD) above normal for age, systemic blood pressure (SBP) <90 mmHg, MAP <70 mmHg, SBP decrease >40 mmHg in adults, or <2 SD below normal for age (26). Sepsis can lead to a low cardiac output, causing tissue hypotension and hypoperfusion. Although the occurrence of sepsis is rare after cardiac surgery, those who develop the condition experience a high mortality (25).

## Post-operative Management

### Fluid Resuscitation

Fluid resuscitation is often the first step in treating patients with low levels of tissue perfusion. The primary reason for treating patients with intravenous fluids is to increase stroke volume through an increase in preload. Following the Frank-Starling principle, increasing LV preload will increase LV stroke volume until an optimal preload is reached, after which the stroke volume will remain relatively constant. However, just 50% of hemodynamically unstable patients respond positively to fluids (volume responsive) (27). For those who are not volume responsive, fluid resuscitation can lead to increased complications. Dynamic tests of volume responsiveness do exist, allowing clinicians to determine the degree to which a patient may be fluid-responsive (28)

### Current Vasoactive Agents

If fluid resuscitation fails to bring the patient's postoperative hemodynamics into a stable range, vasoactive agents are often employed. The most common vasoactive agents are catecholamines. These agents induce sympathetic responses of the autonomic nervous system, triggering the “fight or flight” response. Catecholamines induce cardiovascular effects primarily through α1, β1, β2, and dopaminergic receptors. The activation of α1 adrenergic receptors results in vascular smooth muscle contraction and subsequent increase in SVR. β1 adrenergic receptor activation causes enhanced myocardial contractility through calcium ion channel activation and increased HR. β2 adrenergic stimulation results in an increased calcium ion uptake and vasodilation in skeletal muscle vessels. Lastly, dopaminergic receptor stimulation causes renal and mesenteric vasodilation (29).

Epinephrine is a naturally occurring catecholamine and adrenergic agonist of both α and β receptors. It is a potent vasoconstrictor with inotropic and chronotropic effects. High and prolonged doses can cause cardiac toxicity by damaging arterial walls (29). Although it may be used as an inotropic agent in LCOS, its effects are largely unpredictable (19).

Another catecholamine is norepinephrine, which is primarily an α adrenergic agent, with a modest effect on β adrenergic receptors. It can generate significant vasoconstriction, exhibiting inotropic and chronotropic effects to a much lesser degree. Norepinephrine is generally used for treatment of hypotensive and normovolemic patients experiencing shock (30). Prolonged norepinephrine use, similar to epinephrine, may cause a toxic effect on cardiac myocytes (29).

Dopamine is a naturally occurring catecholamine that acts on dopaminergic and adrenergic receptors to cause a range of hemodynamic effects. At low doses, β receptor responses dominate, resulting in increased contractility and HR with a modest increase in SVR. However, with increasing doses, α1 adrenergic vasoconstriction effects dominate (29). Side effects of dopamine include tachycardia and arrhythmia (31).

Dobutamine is a synthetic catecholamine that is primarily β1 adrenergic. It enhances ventricular contraction with only slight vasodilation effects. It is a potent inotrope that causes mild increases in chronotropic effects, and consequently causes a dose dependent increase in CO. However, it increases myocardial oxygen demand, limiting its clinical use due to risk of ischemia, and may cause ventricular arrhythmias (29, 32).

Another class of vasoactive agents are phosphodiesterase (PDE) inhibitors. PDE inhibitors increase intracellular cyclic adenosine monophosphate which produces inotropic effects as well as systemic and pulmonary vasodilatory effects. Milrinone is the most commonly used PDE inhibitor. Unlike catecholamines, it does not cause increases in myocardial VO_2_ or HR, and is commonly used as a secondary agent when hemodynamic improvement cannot be accomplished with catecholamines (29).

Vasopressin, also known as “antidiuretic hormone,” is stored in the pituitary gland and released after hypotension or pain stimulus, and leads to vasoconstriction, consequently increasing SVR (29). It is not associated with the adverse effects of adrenergic agents, and enables an increase in MAP without adversely affecting CO (33). Another agent used to increase SVR is methylene blue. Methylene blue competes directly with Nitric Oxide (NO) in its ability to activate the enzyme guanylate, which produces cyclic guanosine monophosphate (responsible for smooth muscle relaxation). This reduces the responsiveness of vessels to vasodilators such as NO (34). Both vasopressin and methylene blue can be used to reverse vasoplegia, however methylene blue is recommended as a strategy of last resort (33).

Levosimendan is a relatively new drug that acts as a calcium-sensitizing agent. It increases myofilament sensitivity to calcium, leading to an increase in myocardial contractility without a significant increase in VO_2_. It also causes the opening of potassium channels on vascular smooth muscle, resulting in vasodilation, sometimes leading to undesired hypotension (29, 32). The cardiovascular effects of the vasoactive agents described above are summarized in Table 2.

**Table 2:** Common vasoactive drugs and their effects. Adapted with permission (32, 33).

**Mechanism of action**					**Cardiac**		**Peripheral Vasculature**	
**Drug**	**α1**	**β1**	**β2**	**Dopaminergic**	**Heart rate**	**Contractility**	**Vasoconstriction**	**Vasodilation**
Epinephrine	–/+^*^	++	+	0	+ + ++	+ + ++	+ + ++	+ + +
Norepinephrine	++	++	–	0	+	++	+ + ++	0
Dopamine	–/+^*^	–/+ +^*^	–/+^*^	++	+/+ +^*^	+/+ +^*^	0/+ +^*^	+/0^*^
Dobutamine	–	+ +	+	0	++	++ +/+ + + +^*^	0	++
Milrinone	Phosphodiesterase inhibitors				+	+ + +	0	++
Vasopressin	V1 receptor agonist				0	0	+ + ++	0
Methylene blue	Monoamine oxidase inhibitor				0	0	+ + ++	0
Levosimendan	Calcium-sensitizer				+	+ + +	0	++

* *The ‘/' denotes differences between effects at low vs. high dosages. Typical dosages can be found in Morozowich and Ramakrishna (33) and Hollenberg (32)*.

### Emerging Agents

Although the current repertoire of vasoactive agents is vast, they are not without disadvantages and side effects. The search for new vasoactive agents with capabilities of improving and stabilizing hemodynamics is ongoing, but there are a few recent developments that are promising. These agents will give more control to anesthesiologists, perfusionists, and nurses who are responsible for monitoring and maintaining patient hemodynamics.

One new agent being investigated is istaroxime. It increases sarcoplasmic reticular calcium adenosine triphosphatase and inhibits sodium potassium adenosine triphosphatase activity. This results in an increase in calcium concentration during systole (increasing inotropy) and a rapid decrease in calcium during diastole (increasing lusitropy). These combined effects produce increased systolic and diastolic performance without increasing myocardial oxygen consumption, offering a major advantage over existing inotropes (35).

Another group of vasoactive agents with promising capabilities are cardiac myosin activators. In a trial of patients with stable heart failure and left systolic function, the agent omecamtiv mecarbil demonstrated an ability to increase the duration of left ventricular systole, increasing the ejection fraction (36). This increase improves myocardial efficiency by enhancing ventricular contraction without an increase in myocardial oxygen consumption. Although these agents have not been observed in the immediate intensive care unit environment, they do offer a potential avenue of use in patients with depressed CO after surgery.

## Implications for Lumped-Parameter Modeling

Assessing the efficacy of cardiothoracic surgeries in practice is difficult, relying on past surgeries for guidance, but advances in the field of computational cardiovascular modeling have opened the door to the evaluation of surgeries and medical devices in a virtual setting. One approach to simulate cardiovascular physiology and hemodynamics is by implementing a zero-dimensional lumped-parameter network (LPN). The LPN models the circulatory system as a network of electrical circuit elements, which accounts for the effects of friction, inertia, and vessel compliance with electrical analogs of resistance, inductance, and capacitance, respectively. This gives rise to a system of ordinary differential equations whose solution provides values of hemodynamic variables such as blood pressure and flow rate in different locations of the circulation.

LPN has been used to predict hemodynamic responses under varying circumstances such as surgical intervention, disease conditions, and ventricular assist device support (37). For example, bi-directional Glenn and hemi-Fontan operations for a patient requiring congenital heart defect correction surgery were compared by coupling 3D models of altered geometries with a pre-operative LPN model (1). By using a similar approach, another study developed a framework to study the influence of graft shape in coronary artery bypass graft surgery on local hemodynamics and global circulatory dynamics (2). Furthermore, LPN models have been used for the design and evaluation of cardiovascular assist devices where *in vivo* studies are impractical (38). LPN can also be implemented in a hardware-in-the-loop approach through coupling with a physical experiment; this approach simultaneously captures physical interactions not easily simulated and provides feedback from the physiologic simulation to the experiment (39). Researchers are also developing interactive training modules using LPN models that enable the simulation of cardiovascular diseases and therapies (40).

When constructing a patient-specific LPN physiology model, it is necessary to tune the model to accurately reflect the patient's cardiovascular system. This is accomplished using available clinical data acquired through methods such as cardiac catheterization and magnetic resonance imaging. Standard-of-care patient data acquired prior to surgery are often obtained at different times and when the physiological state of the patient varies due to the influences of factors such as anesthesia. Furthermore, once they have been tuned, LPN parameters are often assumed unchanged in simulations predicting postoperative hemodynamics (1, 2). However, as discussed, the patient physiology changes during the preoperative, intraoperative, and postoperative management periods for several reasons. To advance the state of the art in cardiovascular modeling and improve model prediction accuracy, advanced methods should account for the effects of these factors in simulating postoperative outcomes.

## Conclusion

As discussed, patient cardiovascular physiology in cardiothoracic surgery changes due to many factors: induction of anesthesia, use of cardiopulmonary bypass and cardioplegia, responses to surgical trauma, fluid resuscitation, and administration of vasoactive agents. Without accounting for these factors in lumped-parameter cardiovascular models, the ability of models to predict postoperative hemodynamics will remain limited. The goal of this paper is to provide a summary describing the primary sources of physiologic changes during surgery that affect hemodynamics as the first step in guiding future model improvement; only after recognizing these sources can we know where to direct focus to incorporate their effects quantitatively into models. For example, transient dissipation of anesthesia may be accounted for by integrating pharmacodynamic models to reflect a period of postoperative recovery. As fluid resuscitation and vasoactive agents are available for intentional hemodynamic management, implementing their effects in models may encourage their use in developing surgical strategies, accounting for post-operative management flexibility. With an increasing volume of available data and advances in modeling algorithms, incorporating the complex nature of these factors in cardiovascular models is an area ripe for exploration and pursuit.

## Author Contributions

JK performed literature search and drafted the manuscript, with EK providing the conception and structural design of the paper. EK critically revised the manuscript. JK and EK both performed analysis and interpretation of the literature search results and reviewed the final manuscript.

### Conflict of Interest

The authors declare that the research was conducted in the absence of any commercial or financial relationships that could be construed as a potential conflict of interest.
